# Rationale and design of the Helping Ease Renal failure with *Bupi Yishen* compared with the Angiotensin II Antagonist Losartan (HERBAAL) trial: a randomized controlled trial in non-diabetes stage 4 chronic kidney disease

**DOI:** 10.1186/s12906-015-0830-1

**Published:** 2015-09-08

**Authors:** Wei Mao, Lei Zhang, Chuan Zou, Chuang Li, Yifan Wu, Guobin Su, Xinfeng Guo, Yuchi Wu, Fuhua Lu, Qizhan Lin, Lixin Wang, Kun Bao, Peng Xu, Daixin Zhao, Yu Peng, Hui Liang, Zhaoyu Lu, Yanxiang Gao, Xina Jie, La Zhang, Zehuai Wen, Xusheng Liu

**Affiliations:** Nephrology Center, Guangdong Provincial Hospital of Chinese Medicine, Guangzhou, 510120 China; Key Unit of Methodology in Clinical Research, Guangdong Provincial Hospital of Chinese Medicine, Guangzhou, 510120 China; National Center for Design Measurement and Evaluation in Clinical Research, Guangzhou University of Chinese Medicine, Guangzhou, 510405 China

## Abstract

**Background:**

Chronic kidney disease (CKD) is a global public health problem. Currently, as for advanced CKD populations, medication options limited in angiotensin-converting enzyme inhibitors (ACEi) and angiotensin receptor blockers (ARB), which were partially effective. A Chinese herbal compound, *Bupi Yishen* formula, has showed renal protective potential in experiments and retrospective studies. This study will evaluate the efficacy and safety of *Bupi Yishen* formula (BYF) in patients with CKD stage 4.

**Design:**

In this double blind, double dummy, randomized controlled trial (RCT), there will be 554 non-diabetes stage 4 CKD patients from 16 hospitals included and randomized into two groups: Chinese medicine (CM) group or losartan group. All patients will receive basic conventional therapy. Patients in CM group will be treated with BYF daily while patients in control group will receive losartan 100 mg daily for one year. The primary outcome is the change in estimated glomerular filtration rate (eGFR) over 12 months. Secondary outcomes include the incidence of endpoint events, liver and kidney function, urinary protein creatinine ratio, cardiovascular function and quality of life.

**Discussion:**

This study will be the first multi-center, double blind RCT to assess whether BYF, compared with losartan, will have beneficial effects on eGFR for non-diabetes stage 4 CKD patients. The results will help to provide evidence-based recommendations for clinicians.

**Trial registration:**

Chinese Clinical Trial Registry Number: ChiCTR-TRC-10001518.

## Background

The high incidence of chronic kidney disease (CKD) and associated poor outcomes presents a major public health problem in many developed countries. The prevalence of CKD ranges from 9.4 to 13.0 % in China [1, 2], which is similar to the reported prevalence in North America [3, 4] and Australia [5].

Patients with CKD are at a high risk of cardiovascular events and can gradually develop end stage renal disease (ESRD), with a decreasing estimated glomerular filtration rate (eGFR) of 2 to 20 ml/min/1.73 m^2^ per year [6]. Patients with ESRD require renal replacement therapy to maintain their life, and the rising prevalence of CKD represents an increasing economic burden to many countries. For example, in Taiwan, only 7.7 % of those over the age of 65 are diagnosed with CKD, but 15.9 % of the national health care expenditure is spent treating this condition [7].

The current treatments applied to delay the progression of CKD are limited and only partially effective. Angiotensin-converting enzyme inhibitors (ACEi) and angiotensin receptor blockers (ARBs) are recommended for their antihypertensive and proteinuria-reducing effects [5]. However, ACEi or ARBs can only reduce the risk of ESRD by 50 % [8]. The risks of adverse events including hyperkalemia and acute kidney injury limit the application of ACEi/ARBs in advanced CKD (stages 4 and 5) and elderly patients [9]. Aside from ACEi/ARB and complication management, there are no effective treatments for advanced CKD [5]. Thus, a novel, economical and effective intervention is urgently sought.

In China, Chinese medicine (CM) is widely applied for CKD and an evidence-base application of CM is gradually accumulating. Several large clinical studies have indicated benefit effects of Chinese herbal medicine in early stage CKD patients [10, 11]. However, CM interventions for stage 4 CKD populations are lacking, and further investigations are required to evaluate the risks and benefits of CM in this subgroup population.

*Bupi Yishen* formula (BYF), a Chinese herbal compound, was developed from the classical Chinese prescription Sijunzi decoction, and was modified according to data mining 10,000 CKD stage 4 outpatient records. BYF exhibited a potential renal protective effect in preliminary unpublished observational studies. Twenty-six CKD stage 4 patients were treated with BYF orally once a day based on conventional western therapy. After one-year, eGFR decline was observed in 17 of 26 patients, with a mean reduction of 2.39 (mean: 2.39, standard deviation: 5.59). The decline of eGFR was slower than 6.8 ml/min/1.73 m^2^ per year receiving benazepril in patients with CKD stage 4 that observed in Hou’s study [12].

Components of BYF include *Astragalus mongholicus* (Huangqi), *Codonopsis pilosula* (Dangshen), *Macrocephalae* (Baizhu), *Poria cocos* (Fuling), *Rhizoma dioscoreae* (Shanyao), *Semen coici* (Yiyiren), *Radix polygoni multiflori* (Heshouwu), *Semen cuscutae* (Tusizi), and *Salvia miltiorrhiza* (Danshen). Some of these components have been reported to reduce proteinuria, inhibit renal fibrosis and improve renal function in experimental studies [13–16]. Based on this evidence, a trial was designed to assess the efficacy and safety of BYF for delaying progression in patients with non-diabetes stage 4 CKD. The efficacy of BYF was compared with an Angiotensin II antagonist, Losartan.

## Design and Methods

### Study design

**Fig. 1:**
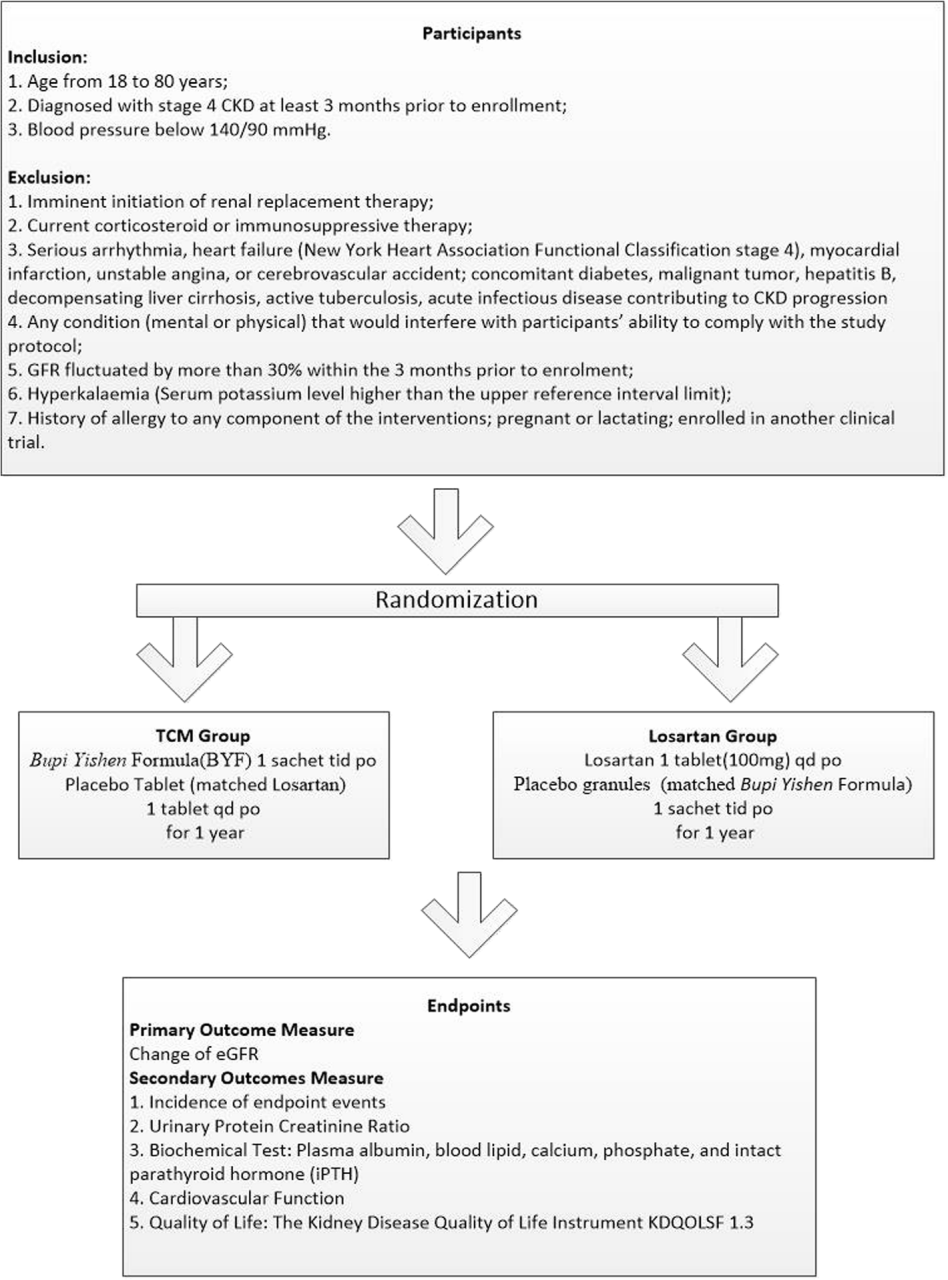
Clinical trial flow diagram

This multi-center, double blind, double dummy, randomized controlled trial (RCT) will be conducted in China (see Fig. 1). Approximately 554 patients will be divided into the CM group or losartan group at a 1:1 ratio.

**Fig. 2:**
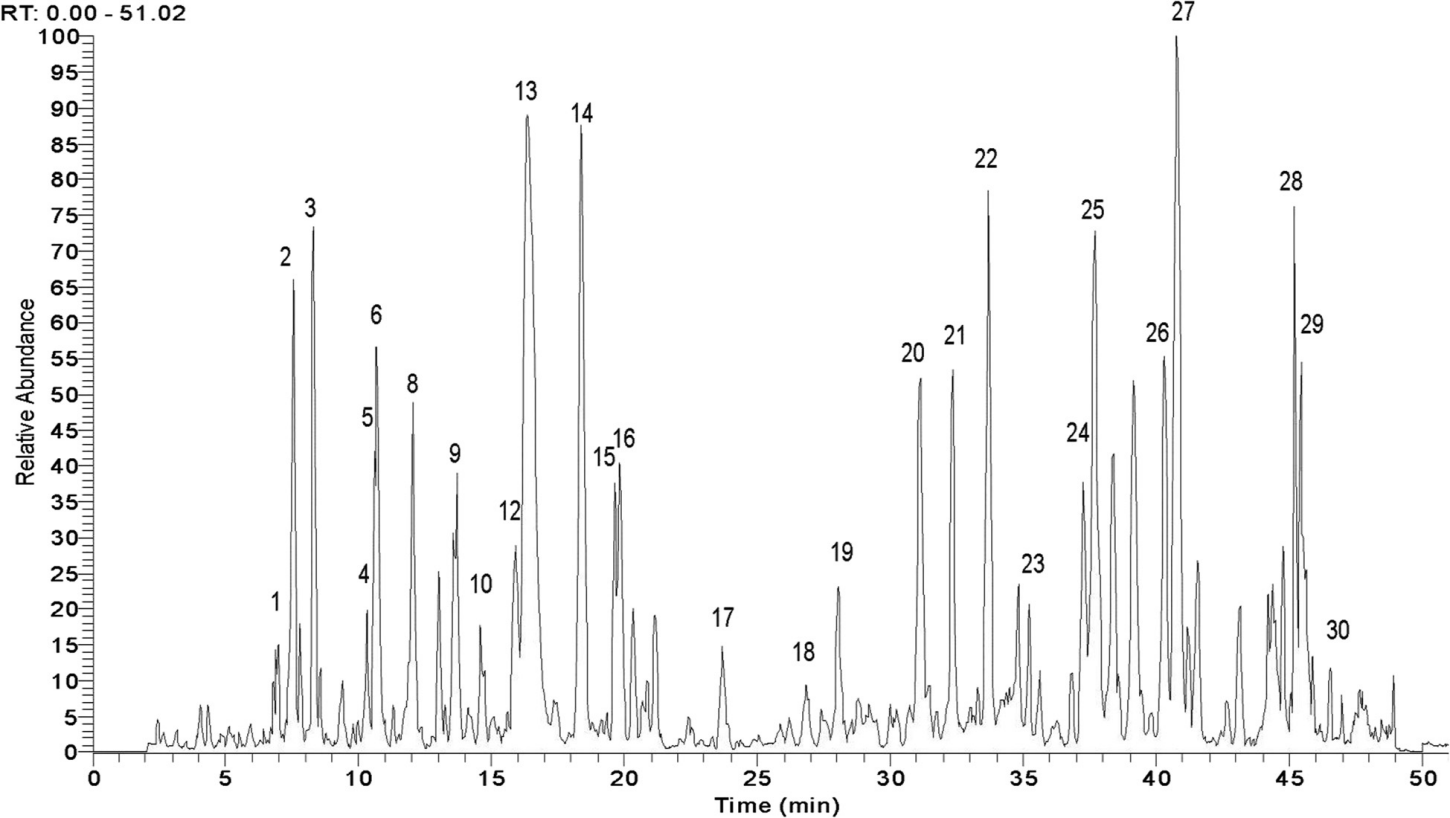
Ultra-performance liquid chromatography-mass spectrometry pattern of Bupi Yishen formula

**Fig. 3:**
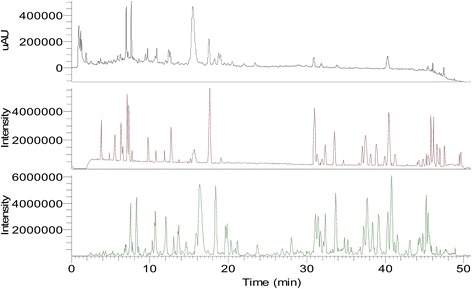
Mass spectra of Bupi Yishen formula (ultraviolet; positive ion mode; negative ion mode)

### Ethics approval and registry

The study will be conducted in accordance with the Declaration of Helsinki, and was approved by the Ethics Committee of Guangdong Provincial Hospital of Chinese Medicine (No. B2010-11-03). The study was registered at the Chinese Clinical Trial Registry (No. ChiCTR-TRC-10001518). Written informed consent will be obtained from each eligible patient.

### Settings

The study will be organized by the Guangdong Provincial Hospital of Chinese Medicine and conducted at 16 centers identified as tertiary level TCM hospitals from different provinces in China.

### Participants

All patients diagnosed with stage 4 CKD and CM syndrome-spleen and kidney qi deficiency (SKQD) will be recruited. Stage 4 CKD is defined as a severe reduction in eGFR of 15–29.9 ml/min/1.73 m^2^ for at least 3 months [17]. SKQD is a CM diagnose based on clinical symptoms, signs, tongue appearance and pulse [18, 19], and was confirmed by two senior physicians.

### Inclusion and exclusion criteria

The participant inclusion criteria are as follows: age from 18 to 80 years; diagnosed with stage 4 CKD at least 3 months prior to enrollment; blood pressure below 140/90 mmHg.

The participant exclusion criteria are as follows: imminent initiation of renal replacement therapy; current corticosteroid or immunosuppressive therapy; serious arrhythmia, heart failure (New York Heart Association Functional Classification stage 4), myocardial infarction, unstable angina, or cerebrovascular accident; concomitant diabetes, malignant tumor, hepatitis B, decompensating liver cirrhosis, active tuberculosis, acute infectious disease contributing to CKD progression, or any condition (mental or physical) that would interfere with participants’ ability to comply with the study protocol; GFR fluctuated by more than 30 % within the 3 months prior to enrollment; hyperkalemia (serum potassium level higher than the upper reference interval limit); history of allergy to any component of the interventions; pregnant or lactating; enrolled in another clinical trial.

### Randomization

Eligible participants will be assigned to each group using blocked randomization, stratified by the participating centers, with a 1:1 allocation ratio using a confidential block size.

Randomization will be performed by the Key Unit of Methodology in Clinical Research (KUMCR) of the Guangdong Provincial Hospital of Chinese Medicine. A computer-generated randomization list created from PROC PLAN in SAS 9.2 (SAS Institute, Cary, NC, USA) will be used. Randomization instructions and blinding codes will be delivered to each participating site via the Interactive Web Response System for Chinese Medicine Trials (IWRS-CMT), a validated web-based randomization system. Each patient will receive a treatment blinding code and all processes will be recorded and saved.

### Blinding

Patients, investigators, monitoring members, outcome assessors, and statisticians will be blinded to group assignment and intervention. The randomization list and blinding codes will remain confidential, and only the KUMCR staff will have access to the randomization list. Blinding will be ensured using the double-dummy method.

### Intervention

Enrollment will be followed by a 3-week run-in period, and then patients will receive BYF plus losartan-matched placebo (CM group) or losartan plus BYF-matched placebo (losartan group) for one year. Patients with unstable BP within the six weeks prior to enrollment that are taking ACEi/ARBs, will be administered other types of antihypertensive agents to reduce BP below 140/90 mmHg.

All Chinese herbs will be obtained from Guangdong Kangmei Pharmaceutical Co. (Guangzhou, Guangdong, China). All herbs will be authenticated by macroscopic and microscopic examination of cross sections, chemical tests and chromatographic analysis. To ensure these herbs are safe for human consumption the agricultural residue screening test for heavy metals, pesticides, and aflatoxins will be performed.

The BYF herbs will be extracted with hot water, then concentrated, spray-dried, and packed in sealed opaque sachets. The entire manufacturing process will comply with Chinese GMP. Sandoz Corp. Ltd. (Zhongshan city, Guangdong, China) will produce the losartan tablet placebos with an identical appearance, smell and taste, packed in exactly the same way.

In the BYF group, patients will be instructed to dissolve a BYF sachet in 150 ml of boiled water and to take this solution orally three times daily and a losartan-matched placebo daily for 1 year. Patients in the losartan group will be instructed to take 100 mg losartan orally once daily and also a placebo sachet dissolved in 150 ml of boiled water three times daily for 1 year.

### Concurrent CKD-related management

All participants will also receive conventional CKD management, including dietary therapy, blood pressure control, management of anemia, regulation of fluid and acid–base disorders, etc., without limitation of dialysis or transplant planning according to the Clinical Practice Guideline [17].

During the intervention period, patients will be advised not to take any other ACEi/ARB or other Chinese medicines with a similar function as the BYF or compound α-ketoacid tablets, and if they do so these patients will be excluded.

### Outcome measurements

#### Primary outcome

The primary outcome is the change in eGFR over 12 months. Decline of eGFR has been widely applied as primary outcome in several trials, such as the Hypertension (AASK) [20], MDRD Study [21] and the Ramipril in Non-diabetic Renal Failure Study (REIN) [22]. The eGFR will be calculated by the CKD-EPI (Chronic Kidney Disease Epidemiology Collaboration) formula [23], and evaluated at baseline, and after 3, 6, 9 and 12 months of the follow-up period.

#### Secondary outcomes

The secondary outcomes include urinary protein creatinine ratio, serum creatinine level, biochemical test, cardiovascular function, quality of life and endpoint events (Table 1).

**Table 1:**
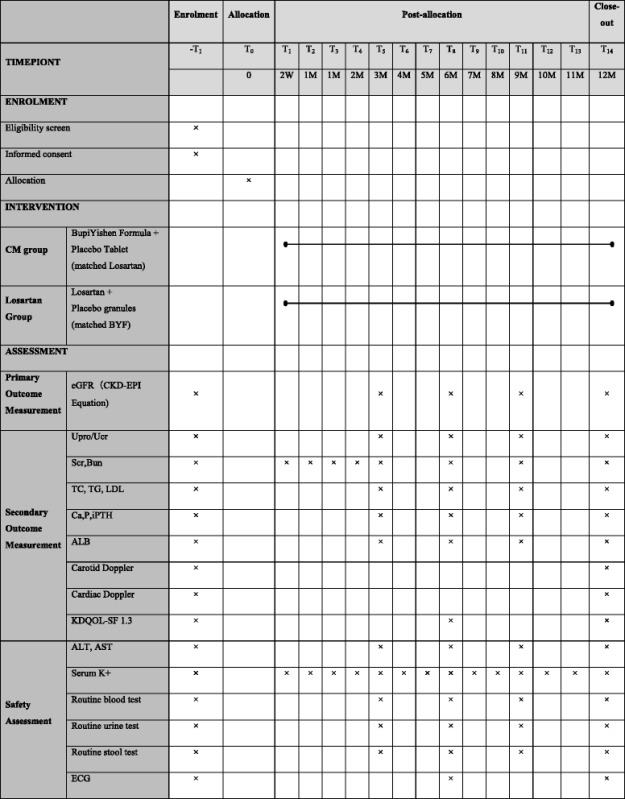
Schedule of study

The urinary protein creatinine ratio will be examined at baseline, and at 3, 6, 9 and 12 months. Serum creatinine level (SCr) and blood urea nitrogen level (BUN) will be measured at baseline, and at 1, 2, 3, 6, 9, and 12 months. Biochemical tests including plasma albumin, triglyceride, cholesterol, calcium, phosphate, and intact parathyroid hormone level (iPTH) will be measured at baseline, and at 3, 6, 9, and 12 months.

We will assess the patient's cardiovascular function by left ventricular ejection fraction (LVEF), interventricular septal thickness (IVST), left ventricular posterior wall thickness (LVPWT), carotid artery intima-media thickness (CIMT) through the Carotid Doppler and Cardiac Doppler, which will be measured at baseline and 12 months.

The Kidney Disease Quality of Life Instrument (KDQOLSF 1.3) scale will be evaluated at baseline and at 6 and 12 months.

Endpoint events are defined as serum creatinine level increasing to double the baseline level, ESRD (requiring permanent dialysis or kidney transplantation), cardiovascular or cerebrovascular events (acute myocardial infarction, heart failure, ischemic cerebral infarction or cerebral hemorrhage verified by typical clinical manifestations, electrocardiogram (ECG) alterations, and imaging and laboratory investigations) or death. The nature and severity of events will be recorded as endpoint events during the follow-up period.

### Safety evaluation

Participants will be asked to report all adverse events (AEs) at each follow up. AEs are defined as any symptom occurring during the intervention period [24]. Expected AEs include dizziness, diarrhea and thirst. Where AEs are reported, clinicians will provide the appropriate treatment in accordance with the study standard operative procedures (SOPs). Laboratory examinations, including routine blood test, urine and stool tests, serum potassium, liver function (AST, ALT), will be monitored at baseline, 3, 6, 9 and 12 months. Serum potassium concentration will be closely monitored at each month. ECG will be reviewed every six month. All AEs will be classified by investigator based on treatment, involving system, severity and temporal correlation with interventions.

Any serious adverse events (SAEs) will be reported, recorded and treated within 24 hours, and reported to the principle investigator and ethics committee for causality assessment. The causality assessment for AEs will be performed according to the WHO-UMC standard [25].

### Sample size calculation

The sample size calculation will be based upon a retrospective analysis of the change in eGFR in patients with stage 4 CKD treated at our hospital between 2003 to 2010, and Hou’s trial published in 2006 [12]. Our retrospective analysis indicated that the mean eGFR decrease was 4.5 ± 4.2 ml/min/1.73 m^2^ per year in patients with stage 4 CKD treated with CM. Hou’s trial reported that the median rate of GFR decrease was around 6.8 ml/min/1.73 m^2^ per year in patients with stage 4 CKD receiving benazepril [12]. PASS 2008 software (NCSS, LLC, Kaysville, Utah, USA) was used to calculate the sample size required for the current study to allow a superiority test to be performed for the BYF group versus the losartan group where 1 ml/min/1.73 m^2^ GFR is considered to indicate a clinically important difference with a 90 % power and a one-tailed significance level of 2.5 %. The study was calculated to require 221 patients per group. Predicting a 20 % lost to follow-up rate, we adjusted the total sample size to 277 for each group.

### Statistical analyses

An independent team of data managers and statisticians from KUMCR, will be responsible for the data management and statistical analysis of this study. The statisticians will be blinded to the randomized allocation of groups. PAWS 18.0 (IBM SPSS Inc., Armonk, New York, USA) and SAS 9.2 (SAS Institute Inc., Cary, USA) statistical software packages will be used to analyze the data.

Prior to analyses, the statistical analysis plan will be written, updated and confirmed after blind data review. The full analysis population is defined as patients randomized to receive at least one treatment dose, with baseline and at least one post-baseline efficacy assessment. The per-protocol population is defined as patients who complete the course of the study without a major protocol violation. The primary outcome analysis is based on full analysis population, intention-to-treat principle and the per protocol population. An intention-to-treat analysis will be applied using the imputation method for missing data. The linear trend at point method will be used to replace missing values. The existing series will be regressed on an index variable scaled 1 to n, and the missing values will be replaced with their predicted values. If the predicted values cannot be computed, the last observation carried forward (LOCF) method will be used for this variable. For analysis of endpoint events, a mixed effect model will also be employed, adjusting for co-variables including baseline characteristics. After reporting descriptive statistics of baseline characteristics for each group, an effectiveness analysis will compare the BYF and losartan groups, and a superiority test, with a 95 % confidence interval for eGFR, will be performed to investigate the clinical difference associated with BYF.

The eGFR will be analyzed using a mixed effect GLM with terms for treatment, time point, center, and some baseline characteristics as co-variables based on the concept of repeated measure analysis or adjusted nominal significance level. The Mann–Whitney U-test will be used to analyze ordinal categorical variables. An analysis of variance (ANOVA) will be used for numerical variables, and a Chi-square test or Fisher exact test will be used for categorical variables.

An analysis of covariance will be used for continuous variables, and the Cochran–Mantel–Haenszel test will be used for categorical variables to adjust for the center effect. Unconditional logistic regression analysis will be used for the curative effect factors analysis. The Kaplan–Meier method will be used to assess the incidence and mean occurrence time of endpoint events. Cox proportional hazards regression will be used to analyze factors influencing the incidence of endpoint events. Subgroup analysis of risk factors including hypertension, proteinuria will be performed. The Fisher exact test will be used to compare the incidence rates of AEs and adverse drug reactions over the course of treatment.

### Termination and withdrawal

Patients failing to complete the clinical observation will be classified as case dropouts. Investigators will contact these patients and attempt to record the reason for dropping out and the last time they took the studied medicine. Participants will be withdrawal from the study in any of the following situations: 1) commencing permanent renal replacement therapy (dialysis >30 days or kidney transplantation); 2) severe infection, hyperkalemia (K^+^ > 6.5 mmol/L), acidosis (TCO_2_ < 13 mmol/L), or heart failure not curable by medication, deterioration of kidney function that demands dialysis, massive hemorrhage of the gastrointestinal tract, serious arrhythmia dependent on anti-arrhythmic drugs, or uremic encephalopathy; 3) severe primary disease such as an active-stage malignant tumor, decompensating liver cirrhosis, or hematopoietic system disease; 4) urinary tract obstruction that requires surgical treatment; 5) death; 6) withdrawal of consent; 7) transfer to a renal unit which is not an active study site.

### Quality control and compliance improvement

This trial will be monitored by contract research organization program managers from, Guangdong International Clinical Research Center of Chinese Medicine (Guangzhou, China), contracted by the research sponsor. The program manager and their team will visit each site to examine trial procedures, ensure data quality, and monitor compliance with the protocol. A data safety monitoring board, consisting of seven independent members, will be established to assess safety events, primary outcomes, and data quality. The department of science research of Guangdong Provincial Hospital of Chinese Medicine will be responsible for trial auditing and inspection for the study.

Measurement of eGFR will be delivered in accordance with the protocol. The investigators will attend a 3-hour training workshop on the protocol and will be provided with a protocol and relevant SOPs. The detection of SCr used to estimate eGFR will be standardized at all sites. All sites will be requested to use the same method, procedure, and reagent when performing SCr detection. The precision performance of SCr will be evaluated using the guidelines in the CLSI Document EP15-A2 published in 2005 by American Clinical and Laboratory Standards Institution (CLSI) [26]. The laboratory method of SCr concentration detection at each site will be validated with a standard substance and will only be accepted if the SCr coefficient of variation is between 1.0 % and 3.0 %.

## Discussion

This multi-center, double blind RCT in stage 4 CKD patients aims to assess the capacity of a Chinese herbal formula BYF to reduce progression of stage 4 CKD. Design of this trial complies with the good clinical practice (GCP) guidelines for evaluating efficacy and safety.

**Chart 1:**
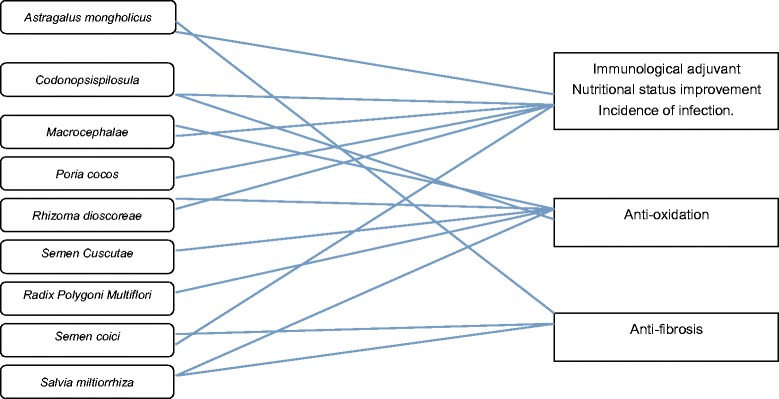
Underlying Multi-compound, multi-target, multi-pathway network of Bupi Yishen formula in retarding progression of chronic kidney disease

In China, herbal formulas processed by water boiling, called decoctions, have been used clinically for thousands of years. These formulas usually consist of several different herbs formulated based on CM theory. Chinese herbal medicine has also been applied specifically to CKD patients for years. The BYF originated from one of the most famous Chinese prescriptions *Sijunzi* decoction, and was modified by senior CM practitioners according to the results of data mining 10,000 CKD stage 4 patients’ medical records. This formula, consisting of nine herbs, has been reported to exert a protective effect in CKD. In this formula, *Astragalus mongholicus* (Huangqi), *Codonopsis pilosula* (Dangshen), *Macrocephalae* (Baizhu), *Poria cocos* (Fuling), *Rhizoma dioscoreae* (Shanyao) may regulate immune functions, improve nutrient status and reduce the incidence of infection [27–31]. *Astragalus mongholicus* (Huangqi), *Salvia miltiorrhiza* (Danshen) and *Semen coici* (Yiyiren) may exert a anti-fibrotic effect [32–34] while *Codonopsis pilosula* (Dangshen), S*emen cuscutae* (Tusizi), *Radix polygoni multiflori* (Heshouwu), *Salvia miltiorrhiza* (Danshen) may reduce oxidative stress [35–38]. The underling mechanism and ratio of each component of *Bupi Yishen* Formula are shown in Chart 1 and Table 2.

**Table 2:** Buipi Yishen formula

Herb	Ratio
*Astragalus mongholicus*	6
*Codono psispilosula*	4
*Macrocephalae*	3
*Poria cocos*	3
*Rhizoma dioscoreae*	3
*Semen Cuscutae*	3
*Radix Polygoni Multiflor*	3
*Semen coici*	4
*Salvia miltiorrhiza*	3

The chemical content of BYF was assessed by Ultra-performance liquid chromatography-mass spectrometry (LC-MS), and the five major chemical components were found to be Salvianolic acid B, Calycosin, Cuscutic acid C, glycerin trioleate and Astragaloside I (Figs. 2 and 3). In 2014, the BYF formula and the methods of producing it were approved and patented as a treatment for CKD by the Chinese State Intellectual Property Office (NO. 2012100298628).

In this trial, the efficacy of BYF will be compared to losartan. In CKD, patients’ GFR diminishes, and the risk of cardiovascular disease increases. ACEI/ARBs are known to exert both renal and cardiac protective effects. Recently, the ROAD study confirmed the benefit of ARB in stage 4 CKD [8]. Therefore, it is appropriate to choose losartan as a positive control rather than placebo. Though Losartan may improve outcomes of CKD patients, more efficacious treatments would still be desirable. Based on the concern above, this study has been designed to compare the effect of CM with the effects of losartan.

The conclusions that may be drawn from the results of this trial are limited by the lack of long-term follow-up and evaluation. However, when the trial ends, those patients that have not reached the endpoints will be admitted to our research-based clinic and continue the optimized CM treatment. All patients will be followed up until they reach the endpoint. Further findings of this trial will be updated.

## Conclusions

This study will be the first double blind RCT to compare the clinical effect of BYF and losartan on eGFR. We will assess the capacity of BYF to reduce the incidence of endpoint events, improve clinical symptoms and quality of life. The trial was carefully designed in collaboration with investigators, clinicians, methodologists, and statisticians. It will be performed rigorously according to ethical and scientific principles. The results of this trial will help to provide evidence-based recommendations for clinicians.

### Trial status

The trial is currently ongoing; we expect patient recruitment to be completed in 2015.

## Abbreviations

ACEI: Angiotensin-converting enzyme inhibitors
ALB: Albumin
ALT: Alanine aminotransferase
ARB: Angiotensin receptor blockers
AST: Aspartate aminotransferase
BP: Blood pressure
BUN: Blood urea nitrogen
Ca: Serum calcium
CAM: Complementary and alternative medicine
CKD: Chronic Kidney Disease
CKD-EPI: Chronic Kidney Disease Epidemiology Collaboration
CVD: Cardiovascular disease
ECG: Electrocardiogram
eGFR: Estimated glomerular filtration rate
eGFR-EPI: Chronic Kidney Disease Epidemiology Collaboration formula
ESRD: End stage renal disease
iPTH: Intact parathyroid hormone
K: Serum potassium
KDQOL-SF: Kidney Disease Quality of Life Short Form
KUMCR: The Key Unit of Methodology in Clinical Research
LDL: Low density lipoprotein
NKF-K/DOQI: National Kidney Foundation Kidney Disease Outcomes Quality Initiative
NYHA: New York Heart Association
P: Serum phosphate
RCT: Randomized controlled trial
ROAD: Renoprotection of Optimal Antiproteinuric Doses
SCr: Serum Creatinine
TC: Total cholesterol
TCM: Traditional Chinese Medicine
TG: Total glycerin trimyristate
Upro/Ucr: Urine protein creatinine ratio
